# Yam-Active Protein Protects Against Cyclophosphamide-Induced Testicular Injury by Suppressing Inflammatory Responses

**DOI:** 10.3390/molecules31091387

**Published:** 2026-04-23

**Authors:** Jiahong Lu, Kaiwen Hao, Yuting Song, Jiaqi Fang, Boyuan Hu, Wei Liu, Ge Hui, Yunfei Xie, Yu Zhao

**Affiliations:** 1 Jilin Ginseng Academy, Changchun University of Chinese Medicine, Changchun 130117, China; 2 College of Food Science and Engineering, Jilin University, Changchun 130062, China; 3 School of Food Science and Technology, Jiangnan University, Wuxi 214122, China

**Keywords:** yam protein, testicular inflammation, cyclophosphamide, spermatogenic dysfunction

## Abstract

Chemotherapy-induced gonadotoxicity severely compromises male fertility, yet effective interventions remain limited. Building on our previous finding that yam protein (YP) modulates the gut-microbiota axis, this study investigated its direct protective role against cyclophosphamide (CTX)-induced testicular injury. Spectral analysis revealed a protein fraction (L-YP) with strong intrinsic fluorescence and optimal cytoprotection against oxidative stress. Proteomic characterization revealed six dominant proteins (YP1–YP6). In vivo experiments demonstrated that L-YP upregulates the expression of tight junction proteins Occludin and ZO-1, restores hormone levels, and modulates inflammatory factors, thereby enhancing the integrity of the blood–testis barrier. Network pharmacology analysis and molecular docking predicted a potential binding affinity between key components such as YP2 and NF-κB p65, which may provide a structural basis for their regulatory role. Further validation at the gene level indicated that YP can improve the local testicular immune microenvironment by modulating the classical TLR4/MyD88/NF-κB inflammatory signaling pathway. These findings suggest that yam protein alleviates chemotherapy-induced testicular damage, potentially through barrier protection and anti-inflammatory mechanisms, indicating its promise as a dietary protective agent.

## 1. Introduction

Yam (*Dioscorea opposita Thunb.*) is a traditional medicinal food [1] with recognized benefits for male reproductive health [2,3]. Although yam extracts have been shown to alleviate spermatogenic disorders [4,5,6], their key bioactive constituents, particularly proteins which constitute 8.40–11.97% of yam [7], remain poorly characterized. The structural and functional properties of yam proteins, as well as their potential mechanism against testicular inflammation (orchitis), are largely unknown. Unlike yam polysaccharides, which primarily exert effects via gut microbiota modulation or surface receptor binding, and small-molecule saponins that may exhibit non-specific distribution, yam proteins possess unique three-dimensional structural domains. These domains confer the potential for specific, high-affinity interactions with intracellular signaling proteins, thus representing a distinct mechanistic avenue for direct intervention in inflammatory cascades.

The aetiology of testicular inflammation is multifaceted. Among the various contributing factors, reproductive toxicity induced by chemotherapeutic drugs is particularly prominent and has become a significant clinical challenge that severely impairs male fertility [8,9]. CTX, a common chemotherapeutic agent [10], is frequently used to establish experimental models of this condition, as its metabolites induce oxidative stress [11], disrupt the blood–testis barrier [12], and trigger testicular inflammation, ultimately impairing spermatogenesis [13,14]. Current interventions, such as the use of glucocorticoids and antioxidants, are often limited by side effects or inadequate efficacy [15], highlighting the need for safer and more effective agents. Consequently, interest in exploring natural bioactive compounds for their potential in mitigating these adverse effects is growing.

On the basis of our previous finding that yam protein modulates the gut microbiota–metabolite axis [16], this study investigates an investigation into its potential direct protective role against CTX-induced orchitis. We aimed to characterize the structural features of the yam protein, evaluate its in vivo efficacy in preserving testicular histology and barrier integrity, as well as in alleviating inflammation, in a CTX-induced murine model, and elucidate its anti-inflammatory mechanism through cytokine profiling and structure–activity relationship analysis, paying particular attention to its interaction with intracellular mediators such as NF-κB p65.

## 2. Results

### 2.1. Extraction Yield, Purity, and SDS-PAGE Analysis of Yam Proteins

The extraction yield and purity of yam proteins from different sources are summarized in Figure S1. The yields for *Dioscorea opposita cv. Lutiegun* protein (L-YP) and *Dioscorea opposita cv. Shatiegun* protein (S-YP) were 3.30% and 3.10%, with corresponding purities of 83.91% and 81.21%, respectively. No significant difference in purity was observed between them.

**Figure 1:**
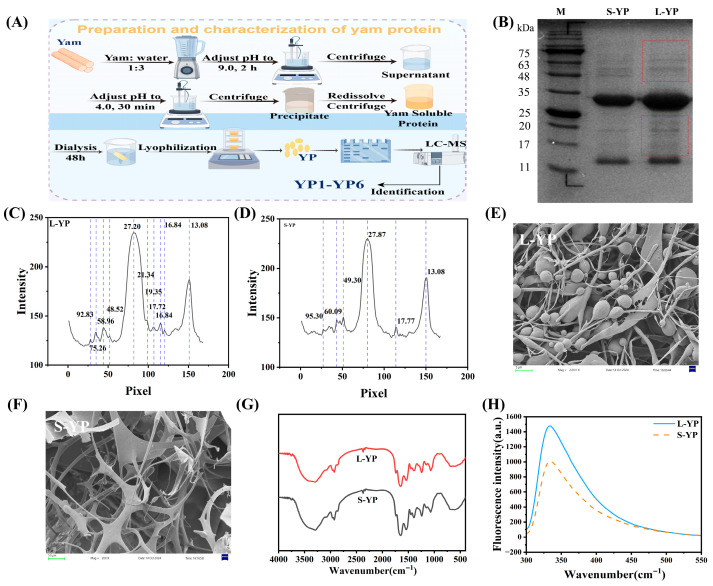
Structural characterization of yam protein. (**A**) Schematic diagram illustrating the extraction and sequential separation of yam protein. (**B**) Sodium dodecyl sulfate-polyacrylamide gel electrophoresis (SDS-PAGE) profile under reducing conditions. (**C**,**D**) Analysis of protein band intensities by SDS-PAGE. (**E**,**F**) Scanning electron microscopy (SEM) images depicting the morphological features at indicated magnifications. (**G**) Fourier-transform infrared (FT-IR) spectroscopy spectra showing characteristic absorption bands. (**H**) Intrinsic fluorescence emission spectra recorded with an excitation wavelength of 290 nm. Specific experimental conditions, protein concentrations, and analytical parameters are detailed in the Section 4.

The extraction and separation process is schematically depicted in Figure 1A. Protein profiles were analyzed by SDS-PAGE (Figure 1B–D). Distinct banding patterns were observed between L-YP and S-YP. A predominant band at approximately 28 kDa (as determined by the standard curve; see Supplementary Materials, Figure S2) was present in both, constituting 23.1% and 14.2% of the total protein in L-YP and S-YP, respectively (Figure 1C,D). Compared to S-YP, L-YP exhibited a broader distribution of protein bands, suggesting a potentially more complex composition.

### 2.2. Characterization and Amino Acid Composition of Different Yam Proteins

The microstructures of L-YP and S-YP were examined by scanning electron microscopy at 500× magnification (Figure 1E,F). Distinct morphological differences were observed: L-YP displayed an intertwined spherical and striped structure, whereas S-YP exhibited a filamentous network.

Fourier-transform infrared (FT-IR) spectroscopy was employed to analyze protein secondary structure (Figure 1G). Both proteins displayed characteristic amide absorption bands. The amide I region (1700–1600 cm^−1^), primarily associated with C=O stretching vibration, was clearly present, confirming their proteinaceous nature [17]. Deconvolution was performed on the amide I region of the infrared spectrum to quantitatively analyze the secondary structures of L-YP and S-YP. The results showed that the relative contents of α-helix, β-sheet, β-turn, and random coil were generally consistent between the two samples, with no significant differences observed (see Supplementary Materials, Table S1). The spectra of the two proteins showed minimal difference.

Intrinsic fluorescence spectroscopy was also conducted (Figure 1H). The fluorescence signal, generated by aromatic amino acid residues (tyrosine, tryptophan, phenylalanine), is sensitive to the local microenvironment and serves as a probe for structural assessment [18]. L-YP exhibited a higher fluorescence intensity compared to S-YP, which may be attributed to a greater content of these fluorophores.

The amino acid compositions of the two yam proteins were analyzed in this study. As shown in Table 1, L-YP had higher contents of essential amino acids for adults (including lysine, leucine, and phenylalanine) and essential amino acids for infants (including histidine and arginine) than S-YP.

### 2.3. In Vitro Antioxidant Activity and Cytocompatibility of Different Yam Proteins

**Figure 2:**
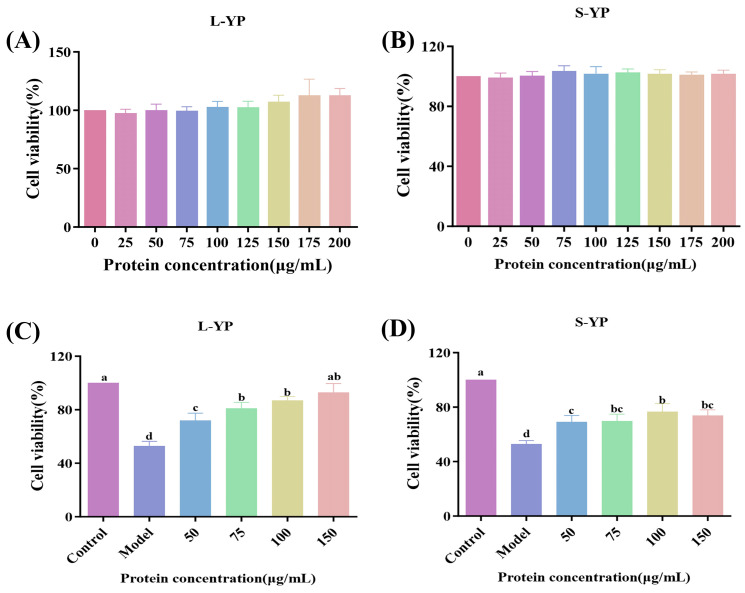
Comparison and screening of the cellular activity of different yam proteins. (**A**,**B**) Cytotoxicity of different yam proteins on TM3 cells; (**C**,**D**) Alleviating effects of different concentrations of different yam proteins on H_2_O_2_-induced damage to TM3 cells. Cells were treated with 0.6 mM H_2_O_2_ (Model group) or co-treated with 0.6 mM H_2_O_2_ and various concentrations (50, 75, 100, and 150 μg/mL) of yam protein. Different lowercase letters indicating statistically significant differences between groups (*p* < 0.05).

The in vitro antioxidant capacity of various yam proteins was evaluated using DPPH and ABTS radical scavenging assays (Figure S3). For DPPH scavenging, L-YP exhibited significantly higher activity than S-YP at concentrations ≥0.5 mg/mL (*p* < 0.05), with an IC_50_ of 0.5 mg/mL and a scavenging rate of 60.84% at 1 mg/mL. Similarly, L-YP demonstrated significantly greater ABTS radical scavenging activity at concentrations ≥0.3 mg/mL (*p* < 0.05). The effects of yam proteins on TM3 cell proliferation were assessed across a concentration range (0–200 μg/mL) (Figure 2A,B). No significant proliferative or cytotoxic effects were observed for either L-YP or S-YP at concentrations up to 200 μg/mL (*p* > 0.05), confirming their cytocompatibility within the tested range.

### 2.4. Effect of Different Yam Proteins on Damaged TM3 Cells

A cell injury model was established by treating TM3 cells with 0.6 mM H_2_O_2_ for 2 h. The effects of L-YP and S-YP on cell recovery were then investigated at final concentrations of 0, 50, 75, 100, and 150 μg/mL.

As shown in Figure 2C,D, a significant therapeutic effect was observed for both L-YP and S-YP. Compared to the model group, a marked improvement in the viability of damaged TM3 cells was achieved at protein concentrations ≥50 μg/mL (*p* < 0.05). The protective effect of L-YP was dose-dependent. Notably, treatment with 75 μg/mL L-YP resulted in 80% cell survival, which was significantly higher than the effect achieved with S-YP. Based on its superior efficacy, L-YP was selected for all subsequent experiments and is hereafter referred to as YP.

### 2.5. Effect of Yam Protein on Oxidative Stress in TM3 Cells

#### 2.5.1. Effect of Yam Protein on Intracellular Reactive Oxygen Species (ROS) Levels

**Figure 3:**
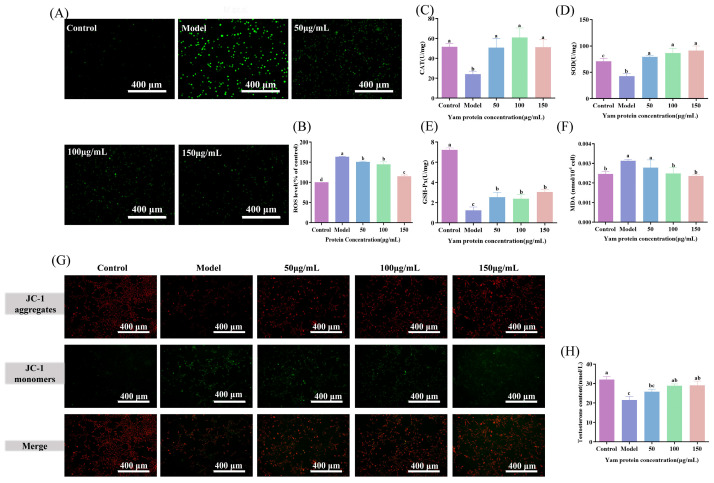
Effects of yam protein on Reactive oxygen species levels, antioxidant enzymes, testosterone content, and mitochondrial membrane potential in H_2_O_2_-damaged TM3 cells. (**A**,**B**) Reactive Oxygen Species (ROS); (**C**) Effects of yam protein on catalase (CAT) enzyme activity in damaged TM3 cells; (**D**) Effects of yam protein on superoxide dismutase (SOD) enzyme activity in damaged TM3 cells; (**E**) Effects of yam protein on glutathione peroxidase (GSH-Px) activity in damaged TM3 cells; (**F**) Effects of yam protein on malondialdehyde (MDA) production in damaged TM3 cells; (**G**) Effect of yam protein on mitochondrial membrane potential in damaged TM3 cells; (**H**) Effect of yam protein on testosterone levels in damaged TM3 cells. Different lowercase letters indicating statistically significant differences between groups (*p* < 0.05).

To explore its anti-inflammatory mechanism, an H_2_O_2_-induced oxidative stress model was established in TM3 cells. H_2_O_2_ treatment significantly elevated intracellular ROS levels. YP administration resulted in a dose-dependent reduction in ROS levels, with the highest dose (YP-H) restoring ROS to near-control values (Figure 3A,B, *p* < 0.05). This direct scavenging of a key inflammatory trigger demonstrates a primary mechanism through which YP alleviates oxidative stress.

#### 2.5.2. Effect of Yam Protein on Antioxidant Enzyme Activities

The impact of YP on the cellular antioxidant defense system was further evaluated. The activities of key antioxidant enzymes (SOD, CAT, and GSH-Px) were significantly suppressed by H_2_O_2_ but were effectively restored upon YP treatment in a concentration-dependent manner, with the highest dose (YP-H) returning SOD and CAT to levels comparable to those of the Control group (Figure 3C–E, *p* < 0.05). Concurrently, the level of MDA was significantly increased by H_2_O_2_ and subsequently reduced by YP, with YP-H lowering MDA to near-control values (Figure 3F, *p* < 0.05). These results indicate that YP not only scavenges ROS directly but also enhances endogenous antioxidant capacity and mitigates oxidative damage.

### 2.6. Effect of Yam Protein on Testosterone Synthesis and Mitochondrial Membrane Potential in H_2_O_2_-Induced TM3 Cells

#### 2.6.1. Effect of Yam Protein on Mitochondrial Membrane Potential

Mitochondrial membrane potential was assessed using JC-1 staining (Figure 3G). Compared to the control group, a reduction in red fluorescence and an increase in green fluorescence were observed in the model group, indicating a loss of membrane potential. This loss was attenuated to varying degrees following intervention with YP, suggesting a protective effect of YP on mitochondrial function.

#### 2.6.2. Effect of Yam Protein on Testosterone Synthesis

The testosterone level secreted by TM3 cells was measured (Figure 3H). A significant decrease was noted in the Model group compared to the Control group (*p* < 0.05). Following YP intervention, the testosterone level was restored, with the highest dose restoring levels to near-control values. This demonstrates the capacity of YP to preserve the endocrine function of TM3 cells under oxidative stress.

### 2.7. Effect of Yam Protein on Organ Indices and Serum Sex Hormone Levels in CTX-Induced Mice

#### 2.7.1. Effect of Yam Protein on Major Organ Indices

**Figure 4:**
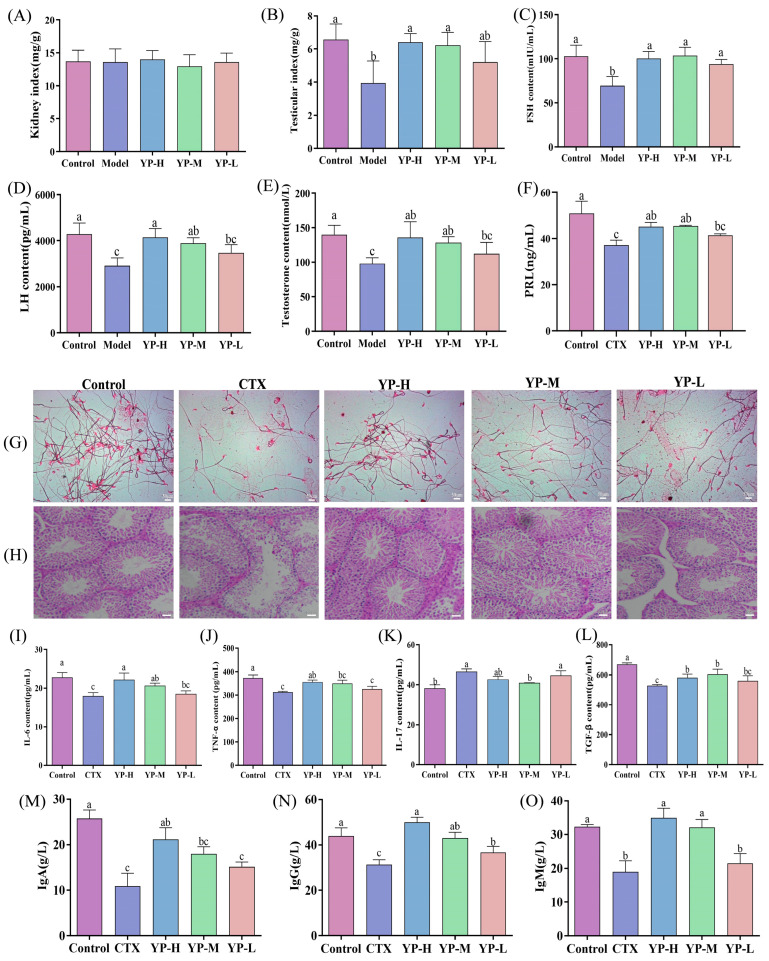
Effects of yam protein on organ indices, blood neutral hormone levels, pathological status, and cytokine levels in CTX-induced mice. (**A**) Kidney index, (**B**) Testis index, (**C**) Follicle-Stimulating Hormone (FSH); (**D**) Luteinizing Hormone (LH); (**E**) Testosterone; (**F**) Prolactin (PRL); (**G**) Sperm morphology; (**H**) Testis morphology; (**I**) Interleukin-6 (IL-6), (**J**) Tumor Necrosis Factor-alpha (TNF-α), (**K**) Interleukin-17 (IL-17), (**L**) Transforming Growth Factor-β (TGF-β), (**M**) Immunoglobulin A (IgA), (**N**) Immunoglobulin G (IgG), (**O**) Immunoglobulin M (IgM). Different lowercase letters indicating statistically significant differences between groups (*p* < 0.05).

The impact of YP on organ indices was evaluated in CTX-induced mice. While kidney weight remained unchanged (Figure 4A, *p* > 0.05), the testicular index was significantly reduced by CTX. This reduction was effectively restored following YP administration (Figure 4B, *p* < 0.05), indicating a protective effect of YP on the reproductive organ.

#### 2.7.2. Effect of Yam Protein on Serum Sex Hormone Levels

Serum levels of FSH, LH, T, and PRL were measured (Figure 4 C–F). CTX induction significantly lowered the concentrations of all four hormones (*p* < 0.05). Treatment with YP resulted in a significant rebound in hormone levels, with the highest dose (YP-H) restoring FSH, LH, T, and PRL to levels approaching those of the Control group (*p* < 0.05). The recovery of testosterone, which is crucial for spermatogenesis [19], alongside prolactin, which modulates gonadal function [20], supports the conclusion that YP mitigates CTX-induced gonadotoxicity. This multi-hormonal restoration may be linked to the amelioration of mitochondrial dysfunction, a known disruptor of energy metabolism and hormone synthesis [21].

### 2.8. Effect of Yam Protein on Sperm Quality and Testicular Histopathological Damage in CTX-Induced Mice

Sperm morphology and count were examined to assess reproductive function (Figure 4G). CTX significantly reduced sperm count and impaired sperm morphology, both of which showed dose-dependent improvement following YP intervention, with the highest dose (YP-H) restoring these parameters to near-control levels. This recovery of key functional sperm parameters directly supports YP’s role in mitigating CTX-induced testicular dysfunction.

Histopathological evaluation of testicular tissue by H&E staining further elucidated this effect (Figure 4H). Compared to the regularly arranged seminiferous tubules in the Control group, the CTX group exhibited severe structural disorganization, spermatogenic cell detachment, and necrosis [22]. YP intervention, particularly at medium and high doses, restored tissue architecture, reduced cellular damage, and increased intratubular sperm presence. This structural restoration provides direct morphological evidence for the testicular protective effect central.

### 2.9. Effect of Yam Protein on Testicular Inflammation and Immune Dysregulation in CTX-Induced Mice

#### 2.9.1. Effect of Yam Protein on Cytokine Expression in Testicular Tissue

To evaluate the local immunomodulatory effect, testicular cytokine levels were measured. CTX induction significantly decreased the concentrations of IL-6, TNF-α, and TGF-β while elevating IL-17 levels (Figure 4I–L, *p* < 0.05). YP administration effectively reversed these alterations in a dose-dependent manner, with the highest dose (YP-H) restoring IL-6, TNF-α, TGF-β, and IL-17 levels to values approaching those of the Control group, which aligns with its proposed role in restoring immune homeostasis.

#### 2.9.2. Effect of Yam Protein on Immunoglobulin (IgG, IgA, IgM) Content in Testicular Tissue

Regarding humoral immunity within the testis, the contents of immunoglobulins (IgA, IgM, IgG) were assessed. CTX induction significantly reduced the levels of all three immunoglobulins (Figure 4M–O, *p* < 0.05). These reductions were notably counteracted by YP treatment in a dose-dependent manner, with the highest dose (YP-H) restoring IgA, IgM, and IgG levels to near-control values (*p* < 0.05). This modulation of both inflammatory mediators and local immunoglobulin levels demonstrates the capacity of YP to target and restore the testicular immune microenvironment, which is a key mechanism for mitigating injury.

### 2.10. Effect of Yam Protein on the Integrity of the Testicular Barrier in CTX-Induced Mice

**Figure 5:**
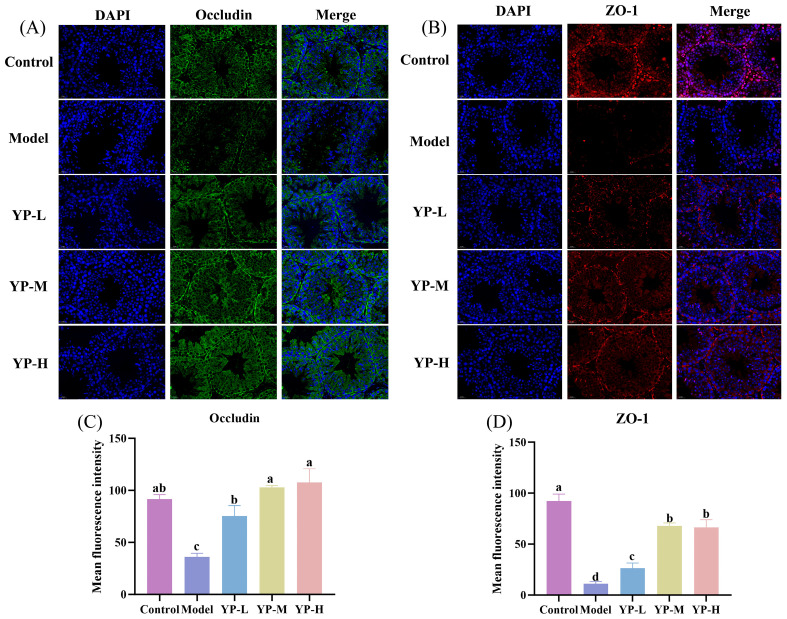
Effect of yam protein on cyclophosphamide-induced testicular permeability in mice. (**A**,**C**) Occludin; (**B**,**D**) Zonula Occludens-1 (ZO-1). Different lowercase letters indicating statistically significant differences between groups (*p* < 0.05).

The integrity of the blood–testis barrier (BTB), which is essential for testicular immune privilege and is compromised during inflammation [23], was investigated to directly evaluate the “barrier-protective” mechanism proposed in our hypothesis. Immunofluorescence analysis revealed that CTX significantly reduced the expression of key BTB proteins, Occludin and ZO-1, compared to the Control group (Figure 5A–D, *p* < 0.05). YP intervention at various concentrations significantly restored the fluorescence intensity of Occludin and ZO-1, with the highest dose (YP-H) returning protein expression to levels comparable to those of the Control group (*p* < 0.05). These results demonstrate that YP mitigates CTX-induced damage to the BTB, providing direct structural evidence for its role in preserving testicular barrier function and immune privilege, which is a central component of its hypothesized protective mechanism against inflammation.

#### 2.10.1. Separation of Yam Proteins by Two-Dimensional Gel Electrophoresis

**Figure 6:**
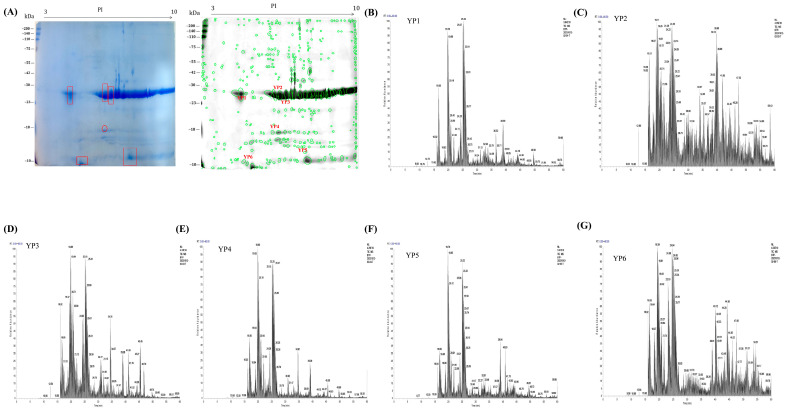
Characterization of yam protein by two-dimensional electrophoresis and mass spectrometry. (**A**) Two-dimensional electrophoresis gel image of yam protein and corresponding protein spots; (**B**–**G**) Six major protein spots (YP1–YP6) were identified from the two-dimensional electrophoresis gel, along with their corresponding mass spectra.

To establish a compositional basis for the observed protective effects, the active YP was separated by two-dimensional gel electrophoresis (Figure 6A). Six well-resolved and representative protein spots (YP1–YP6) were subsequently excised from the gel for further analysis.

#### 2.10.2. Data Alignment and Analysis of Protein Identification Results from Mass Spectrometry

The excised protein spots (YP1–YP6) were subjected to mass spectrometric identification (Figure 6B–G). Database matching against a yam transcriptome identified them as cycloartenol-C-24-methyltransferase (YP1), mitochondrial ornithine aminotransferase (YP2), dioscorin precursor (YP3), tuber lectin 1 (YP4), histone-like protein (YP5), and embryo-specific protein ATS3A (YP6) (Table S3). The presence of known bioactive proteins like dioscorin and a key mitochondrial enzyme links the composition of YP to potential regulatory functions.

### 2.11. Network Pharmacology

**Figure 7:**
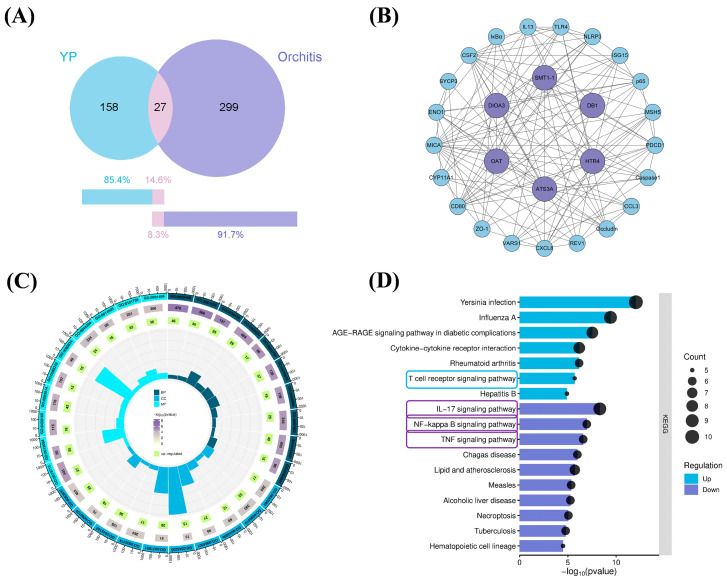
Construction of Yam Protein Target Gene Sets, Protein–Protein Interaction (PPI) Networks, Target Gene Ontology Enrichment Analysis, and KEGG Pathway Mapping. (**A**) Construct a common target gene set for yam protein and orchitis; (**B**) Interactions among target genes; (**C**) GO enrichment analysis; (**D**) KEGG pathway mapping.

Based on the transcriptome data of yam, 185 core proteins were screened and compared with 326 disease targets related to “orchitis” from the GeneCards database, resulting in the identification of 27 potential overlapping targets (Figure 7A). Based on established protein–protein interaction data, a PPI network was constructed for the identified overlapping targets (Figure 7B). Further analysis revealed that these targets include key inflammation- and immune-related molecules such as TLR4, MyD88, NF-κB p65, and NLRP3, indicating that these targets may be involved in the anti-inflammatory effects of YP, warranting further experimental investigation. Subsequent KEGG pathway enrichment analysis indicated that the relevant targets were significantly enriched in the T cell receptor signaling pathway (upregulated), as well as the IL-17 signaling pathway, NF-κB signaling pathway, and TNF signaling pathway (downregulated) (Figure 7C,D). Based on these predictions, we selected the identified targets (TLR4, MyD88, NF-κB p65, NLRP3) for subsequent molecular docking studies to explore potential interactions at the structural level.

### 2.12. Molecular Docking and Molecular Dynamics Analysis

**Figure 8:**
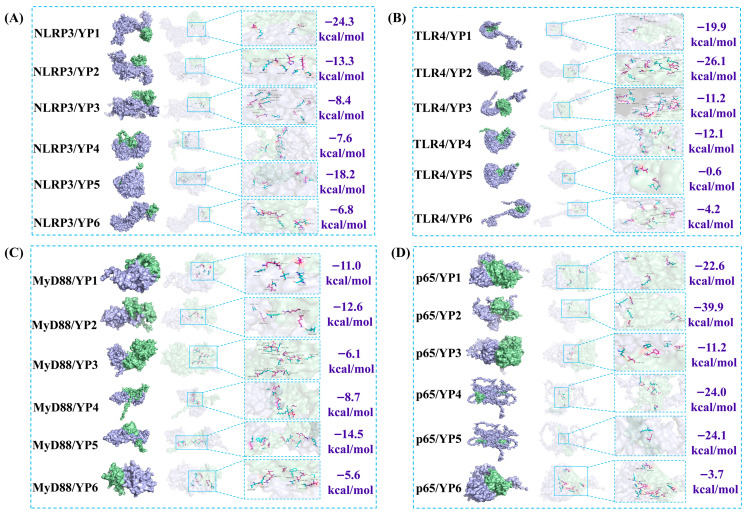
Molecular docking. (**A**) Molecular docking and binding energy of NLRP3 with six major proteins; (**B**) molecular docking and binding energy of TLR4 with six major proteins; (**C**) molecular docking and binding energy of MyD88 with six major proteins; (**D**) molecular docking and binding energy of p65 with six major proteins. Purple indicates the target protein, and green indicates YP1–YP6.

To investigate the primary molecular mechanism of protein action, molecular docking was performed to explore the potential binding modes between YP components and the target proteins. As shown in Figure 8, YP2 exhibited predicted interactions with residues including GLU-271, ALA-246, and LEU-248 of NLRP3, p65, TLR4, and MyD88. The calculated binding energies for all six YP components with their respective targets were more favorable than −4 kcal/mol, which suggests a potential binding tendency that warrants further experimental validation. Among them, YP2 showed the most favorable predicted the highest binding affinity with p65 [24]. It should be noted, however, that the molecular docking results merely indicate potential binding affinity at the structural level; the actual bioactive entity in vivo, whether it be intact YP2, its digestion-derived peptide fragments, or indirect systemic mediators, remains to be identified.

**Figure 9:**
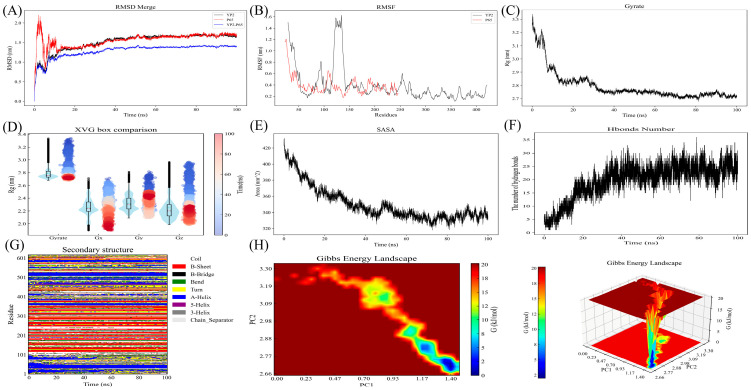
Molecular dynamics. (**A**) Root Mean Square Deviation; (**B**) Root Mean Square Fluctuation; (**C**,**D**) Radius of Gyration; (**E**) SASA; (**F**) Hydrogen Bond; (**G**) Secondary structure; (**H**) Free energy landscape diagram.

Given that semi-flexible docking does not account for protein flexibility, environmental factors such as temperature and pressure, or solvent effects, molecular dynamics simulation was performed to assess the dynamic behavior of the YP2-p65 complex over 100 ns. As shown in Figure 9A–H, the RMSD of the complex remained within 1.4 nm and stabilized around 1.3 nm after 20 ns, indicating that the system reached a relatively stable state under the simulated conditions. RMSF analysis showed higher flexibility in YP2 (particularly residues 100–150), while p65 maintained greater conformational stability. The Rg fluctuated between 2.7 and 3.3 nm with minimal variation after 20 ns, suggesting that the complex maintained a compact conformation throughout the simulation. Secondary structure analysis revealed no major alterations over time, and SASA remained stable between 320–420 nm^2^ from 40–100 ns. Hydrogen bond analysis indicated consistent intermolecular interactions between YP2 and p65 throughout the simulation. Collectively, these parameters suggest that the YP2-p65 complex remained conformationally stable during the 100 ns simulation, providing structural insights into their potential interaction mode.

The Gibbs free energy landscape, constructed using principal component analysis, is shown in Figure 9H. The sampled conformations were predominantly concentrated within low-energy basins (blue and green regions), indicating that the complex adopted one or a few major conformational states during the simulation. This observation is consistent with the other stability parameters described above and supports the notion that the YP2-p65 interaction interface remained relatively stable under the simulated conditions.

### 2.13. PCR Analysis of the Regulatory Effect of YP on Core Genes in Testicular Inflammation

**Figure 10:**
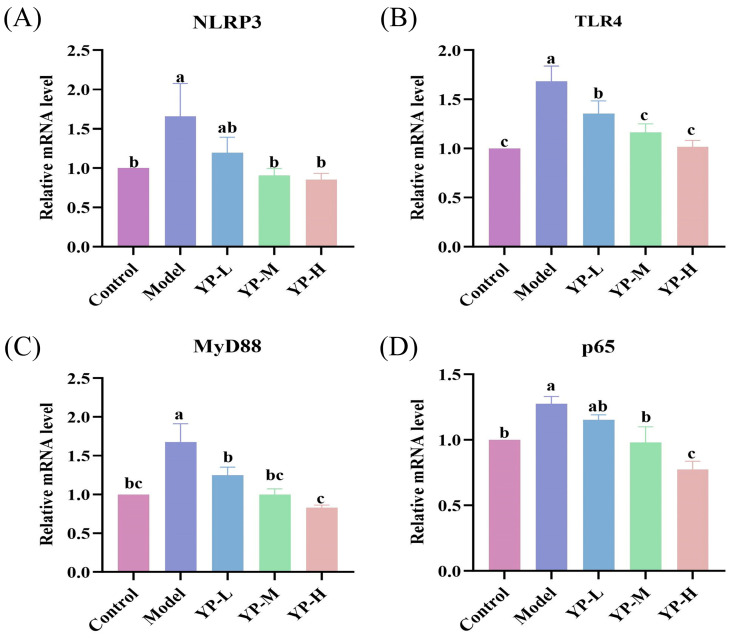
Effects of yam protein on cyclophosphamide-induced inflammatory-related RNA expression in mice testes. (**A**) NLRP3, (**B**) TLR4, (**C**) MyD88, (**D**) p65. Different lowercase letters indicating statistically significant differences between groups (*p* < 0.05).

To validate the aforementioned bioinformatic predictions and molecular simulation results, we performed PCR analysis on key genes in testicular tissues. As shown in Figure 10A–D, YP treatment markedly suppressed the CTX-induced overexpression of NLRP3, TLR4, MyD88, and p65 genes. These experimental findings align with the potential targets identified through network pharmacology and the binding patterns suggested by molecular docking, further confirming that YP can ameliorate the testicular immune microenvironment by modulating the canonical TLR4/MyD88/NF-κB inflammatory signaling pathway.

## 3. Discussion

Yam (*Dioscorea opposita Thunb.*) has long been recognized as a medicinal and edible plant, with its protein components (YP) considered key contributors to its physiological activity. However, the precise structure–activity relationship and the direct mechanism by which YP exerts protective effects on the reproductive system remain unclear. This study systematically demonstrates the protective effect of yam protein against CTX-induced orchitis. Moreover, it provides a multi-dimensional elucidation of its multitarget mechanisms, encompassing structural characterization, cytoprotective effects, in vivo validation in animal models, molecular pathway exploration, and identification of key active constituents. These findings offer new candidate agents and a theoretical foundation for the development of natural protein-based strategies for male reproductive protection.

Normal testicular function depends on precise endocrine regulation, a stable immune microenvironment, and an intact blood–testis barrier [25]. Chemotherapeutic agents such as CTX disrupt this delicate system by inducing oxidative stress and inflammatory responses, ultimately leading to spermatogenic dysfunction and hormonal imbalance [14]. In this study, in vitro experiments revealed that L-YP and S-YP, designated based on their respective soil origins (loess and sandy), exhibited distinct activities, with L-YP showing superior antioxidant effects under our experimental conditions. L-YP significantly alleviated H_2_O_2_-induced oxidative damage in TM3 cells, restoring mitochondrial membrane potential and testosterone secretion. These findings are consistent with those of Yu et al. [5], who reported that a cold extract of yam could modulate oxidative stress markers such as SOD and 8-OHdG in H_2_O_2_-treated TM3 cells, although the specific active constituents were not identified. In contrast, the present study directly validates the effects of yam protein, particularly L-YP, and offers preliminary insight into its potential mechanism. To explore the structural basis for these functional differences, the amino acid compositions of L-YP and S-YP were analyzed. It was found that L-YP contained a higher abundance of aromatic amino acids (such as phenylalanine and tyrosine) compared to S-YP. Given that aromatic amino acids are the primary source of intrinsic protein fluorescence, this compositional difference may contribute to the distinct fluorescence spectral characteristics observed for L-YP. Furthermore, the higher content of aromatic amino acids likely endows L-YP with enhanced free radical scavenging capacity, which, together with its more complex protein band profile, may provide a structural basis for its superior cytoprotective effects. Together, these findings establish both the bioactivity and structural basis for further in vivo application of L-YP.

In mice models of CTX-induced orchitis, we validated the systemic protective efficacy of yam protein (YP, specifically the selected L-YP). YP exerted a marked protective effect on testicular tissue architecture, restoring the testicular index, improving sperm count and morphology, and reversing pathological damage to the seminiferous tubules. It also effectively modulated serum sex hormone levels (FSH, LH, T, and PRL). These findings directly confirm the capacity of YP to counteract CTX-induced gonadotoxicity at the whole-animal level. Its activity extends beyond local testicular tissue repair to involve the regulation of hypothalamic–pituitary–gonadal axis function. These findings are consistent with previous reports: Pierre Watcho et al. demonstrated that Helichrysum odoratissimum (Asteraceae) alleviated CTX-induced reproductive toxicity by regulating sex hormone levels [26]. Similarly, Wang et al. reported that the peptides humanin and MOTS-c mitigated chemotherapy-induced reproductive injury by improving testicular morphology [27], which aligns with the outcomes of the present study. Further investigation revealed that CTX compromised the expression of key tight junction proteins (Occludin and ZO-1) within the blood–testis barrier, an effect that was effectively reversed by YP intervention. The blood–testis barrier constitutes the physical foundation of the testicular immune environment, and its compromised integrity is a key event in inflammatory cell infiltration and autoimmune attack [28]. The protection of this barrier by YP provides a direct structural basis for the maintenance of a stable testicular microenvironment.

To further elucidate the key material basis and molecular targets underlying the protective effects of YP, this study integrated network pharmacology prediction with computational biology validation. Network pharmacology analysis, based on the laboratory’s yam transcriptome data and known targets associated with orchitis, revealed that the major components of YP (YP1–YP6) exhibit strong binding potential with core inflammatory targets, including TLR4, MyD88, NF-κB p65, and NLRP3. KEGG enrichment analysis further showed that these targets were significantly enriched in signaling pathways such as TNF, IL-17, and NF-κB. These pathways play well-defined roles in the pathological progression of orchitis: the TNF and IL-17 pathways are key drivers of local inflammatory responses and immune cell infiltration in the testes [29], whereas the NF-κB pathway serves as a central hub for inflammatory regulation, and its sustained activation can lead to a cytokine storm and exacerbate tissue damage [30]. Thus, the network pharmacology predictions suggest that YP may achieve multitarget intervention in orchitis by coordinately regulating these closely interconnected inflammatory and immune networks. These predictive results are not only consistent with the experimentally observed normalization of NF-κB p65 phosphorylation and downstream cytokine levels (TNF-α, IL-6, and IL-17) by YP, but also further imply that YP may participate in multitarget, systemic intervention in the pathological process of orchitis through synergistic modulation of these functionally linked inflammatory and immune networks.

Following the acquisition of these leads, we further validated the key interactions through molecular docking and dynamics simulations. The results revealed that mitochondrial ornithine aminotransferase (YP2) exhibited a predicted stable binding mode with NF-κB p65, which exhibited favorable structural stability in the dynamics simulations. These findings offer a plausible structural rationale for the experimentally observed inhibition of the NF-κB pathway by YP and implicate YP2 as a candidate contributor to this activity. As an upstream regulator of inflammatory pathways, NF-κB can influence the activity of multiple downstream molecules upon inhibition, including TNF and IL-17 [31]. Together, these computational and bioinformatic findings are consistent with each other and suggest that YP, with YP2 as a potential key contributor, may help alleviate CTX-induced testicular injury through modulation of the NF-κB-associated inflammatory network.

To validate the bioinformatic and computational predictions described above, we examined the gene and protein expression changes of key targets in testicular tissue by PCR and Western blot (see Figure S4, Supplementary Materials). The results showed that YP intervention significantly downregulated the CTX-induced overexpression of genes such as TLR4 and MyD88. These findings are consistent with the target predictions from network pharmacology and the molecular docking results. As a central hub for inflammatory responses, the NF-κB pathway plays a pivotal role in orchestrating immune signaling. Upon activation, it not only directly promotes the expression of proinflammatory cytokines such as TNF-α and IL-6 but also triggers the NLRP3 inflammasome, thereby establishing an amplified inflammatory cascade that exacerbates tissue damage [32]. Similarly, previous studies have demonstrated that natural bioactive compounds such as allicin can simultaneously downregulate key inflammatory mediators, including IL-1β, IL-18, and NLRP3, by inhibiting the NF-κB pathway [33]. In the present study, YP intervention effectively reduced CTX-induced phosphorylation of NF-κB p65 and the levels of its downstream proinflammatory factors (TNF-α, IL-6, and IL-17), while also significantly suppressing the expression of key inflammasome components such as NLRP3 and Caspase-1. This coordinated inhibitory effect suggests that, by targeting the upstream NF-κB hub, YP may simultaneously regulate cytokine production and inflammasome activation, thereby mitigating testicular immune–inflammatory imbalance through multiple mechanisms. Together, these findings substantiate that YP can modulate the testicular immune microenvironment by inhibiting the canonical TLR4/MyD88/NF-κB inflammatory pathway.

It is also pertinent to consider the relationship between the direct testicular protection demonstrated here and the gut-mediated effects identified in our previous investigation. We previously reported that yam protein ameliorates CTX-induced intestinal immunosuppression by modulating the gut microbiota and its metabolites [16]. In light of the emerging concept of the gut–testis axis, wherein microbial metabolites and gut-derived immune signals can influence testicular homeostasis, it is conceivable that the in vivo benefits of YP reflect a combination of direct testicular action and indirect, microbiota-dependent mechanisms. The in vitro cytoprotective data and the computationally predicted YP2–NF-κB p65 interaction unequivocally support a direct component. Nevertheless, a contribution from gut-derived factors that enter the systemic circulation and reach the testicular microenvironment cannot be excluded. Future studies incorporating fecal microbiota transplantation, antibiotic-induced microbiota depletion, or germ-free models will be instrumental in delineating the relative contributions of direct versus gut-mediated pathways.

While the present findings provide a comprehensive foundation, several methodological considerations merit attention. First, the in vivo observations derive from a single mouse strain (ICR); extension to additional genetic backgrounds would help establish broader translational relevance. Second, the use of direct pharmacological inhibitors of the TLR4 pathway will be valuable to delineate the precise hierarchy of YP’s molecular targets. Third, the computationally predicted YP2–NF-κB p65 interaction awaits direct biophysical confirmation through techniques such as surface plasmon resonance or isothermal titration calorimetry. Fourth, although our in vitro experiments in TM3 Leydig cells established the protective effects of YP on oxidative stress and testosterone secretion, this model does not fully recapitulate the Sertoli-cell-based architecture of the blood–testis barrier. Direct assessment of YP’s effects on Sertoli cell barrier function would further strengthen the mechanistic understanding of its protective effects. Future studies incorporating Sertoli cell lines or co-culture models will therefore be valuable to directly evaluate transepithelial electrical resistance and junctional dynamics. Fifth, the translational relevance of dietary intervention strategies based on yam protein requires further investigation. Although our in vivo data demonstrate protective effects of orally administered YP in a murine CTX-induced orchitis model, extrapolation to clinical settings involving human chemotherapy patients remains uncertain. Key translational questions, including the optimal oral dosage regimen, gastrointestinal stability and absorption of YP, and potential interactions with chemotherapeutic agents, have not been addressed. Moreover, the development of yam-derived products as functional food components entails additional practical considerations beyond the scope of this study, such as scalable extraction and purification protocols, formulation stability, sensory attributes, and cost-effectiveness. Future studies integrating pharmacokinetic profiling, long-term safety evaluation, and food matrix compatibility will be essential to bridge the gap between these preclinical findings and real-world applications.

In summary, this study systematically elucidates the multifaceted protective mechanisms of YP against CTX-induced testicular injury, operating through mitigation of oxidative stress, restoration of the blood–testis barrier and hormonal axis, and suppression of the NF-κB/NLRP3 inflammatory axis. Notably, the key component YP2 was predicted to exhibit favorable binding to NF-κB p65, offering a molecular basis for this activity. Collectively, this work clarifies the role of yam protein in reproductive protection across multiple dimensions and establishes a scientific basis for its potential application as a functional protein resource to alleviate chemotherapy-induced gonadotoxicity.

## 4. Materials and Methods

### 4.1. Materials

The *Dioscorea opposita cv. Lutiegun* and *Dioscorea opposita cv. Shatiegun* were purchased from Wen County, Jiaozuo City, Henan Province, and Li County, Baoding City, Hebei Province, respectively. TM3 cells were procured from the Shanghai Cell Bank, Chinese Academy of Sciences (Shanghai, China). All ELISA kits (for IL-6, IgG, IgM, IgA, TNF-α, TGF-β, IL-17) and antibodies (against ZO-1, Occludin) were obtained from Shanghai Enzyme-linked Biotechnology Co., Ltd. (Shanghai, China) and Proteintech Co., Ltd. (Wuhan, China), respectively. CTX was acquired from Sigma-Aldrich (St. Louis, MO, USA). The TM3 cells used in this study were purchased from the Shanghai Institute of Chinese Academy of Sciences, with the catalog number 3101MOUGNM24.

### 4.2. Preparation of Yam Protein

The proteins from the two yams (L-YP, S-YP) were extracted and prepared following the methods outlined in previous studies [16]. The extracts were lyophilized and weighed to calculate the yield. The lyophilized powder was reconstituted in ap-propriate buffer, and protein concentration was determined using a BCA assay kit. Absorbance was measured at 562 nm, and protein concentration was calculated from a bovine serum albumin standard curve. The purity of the lyophilized powder was expressed as the percentage of total protein mass relative to the dry powder mass (*w*/*w*). SDS-PAGE was performed to compare the protein profiles of the two yam samples.

### 4.3. Microscopic Morphology of Yam Protein

The microstructure of yam protein was observed using scanning electron microscopy (SEM) at an accelerating voltage of 3 kV. Freeze-dried samples were sputter-coated with gold prior to imaging, following a modified method based on Hu et al. [34].

### 4.4. Fourier Infrared Spectra of Yam Protein

FT-IR spectra of yam protein were recorded from 400 to 4000 cm^−1^ using the procedure described by Pan et al. [35]. Briefly, samples were dried to constant weight, mixed with KBr, and ground into pellets for measurement. Fitting was performed using Omnic 8.2 software. The spectral region of 1600–1700 cm^−1^ was selected and subsequently subjected to deconvolution using PerkFit 4.1 software. A second derivative was applied for curve fitting to calculate the relative content of each secondary structure component.

### 4.5. Fluorescence Spectrometry of Yam Proteins

Endogenous fluorescence spectra were obtained with an F-7000 fluorescence spectrophotometer according to Fang et al. [36] with minor modifications. Protein solutions (0.25 mg/mL) were excited at 290 nm, and emission was scanned from 300 to 550 nm.

### 4.6. Amino Acid Composition Analysis

Samples (approximately 0.15 g) were hydrolyzed in 10 mL of 6 mol/L HCl containing 0.1% phenol in evacuated and sealed ampoules at 110 °C for 24 h. After cooling, the hydrolysate was transferred to a 50 mL volumetric flask and diluted to volume with ultrapure water. An aliquot (1 mL) of the hydrolysate was filtered, and 500 μL of the filtrate was evaporated to dryness under nitrogen. The residue was reconstituted in 200 μL of 0.1 mol/L HCl. Subsequently, 20 μL of norleucine (internal standard), 100 μL of triethylamine-acetonitrile solution, and 100 μL of phenyl isothiocyanate-acetonitrile solution were added sequentially. The mixture was vortexed and allowed to stand at room temperature for 1 h. Then, 400 μL of n-hexane was added, vortexed, and left for 15 min at room temperature. The lower layer was collected, filtered, and 100 μL of the filtrate was diluted with 400 μL of ultrapure water. A 10 μL aliquot was injected for analysis. Chromatographic analysis was performed on a Waters 1525 HPLC system equipped with a Waters 2998 photodiode array detector (Waters, Milford, MA, USA). The separation was carried out at a column temperature of 40 °C with a flow rate of 0.70 mL/min using gradient elution. Detection wavelength was set at 254 nm with spectral scanning from 210 to 400 nm.

### 4.7. Screening of Yam Protein

TM3 cells were maintained in DMEM/F12 medium supplemented with 10% FBS and 1% penicillin/streptomycin at 37 °C under 5% CO_2_. To evaluate the alleviating effect of yam proteins, cells were injured with 0.6 mM H_2_O_2_ and then treated with different concentrations (50–100 μg/mL) of L-YP or S-YP. Cell viability was measured using the CCK-8 assay. Based on the screening results, L-YP was selected for all subsequent experiments and is hereafter referred to as YP.

### 4.8. In Vitro Cellular Experiments

All in vitro assays described below were performed in three independent replicates, unless otherwise specified.

#### 4.8.1. Mitochondrial Membrane Potential Measurement

Mitochondrial membrane potential was assessed using JC-1 staining. TM3 cells were treated with H_2_O_2_ and then incubated with different concentrations (50–150 μg/mL) of yam protein for 24 h. After staining, cells were observed under a fluorescence microscope, and fluorescence intensity was quantified using ImageJ 1.8.0 software.

#### 4.8.2. Determination of Reactive Oxygen Species (ROS)

Intracellular ROS levels were measured using a DCF-DA assay kit. Fluorescence was recorded at excitation/emission wavelengths of 500 nm and 525 nm, respectively.

#### 4.8.3. Measurement of Cellular Oxidative Stress Biomarkers

The levels of malondialdehyde (MDA), along with the activities of glutathione peroxidase (GSH-Px), superoxide dismutase (SOD), and catalase (CAT), were evaluated in TM3 cells according to the guidelines provided by the manufacturer (Nanjing Jianjian Bioengineering Institute, Nanjing, Jiangsu, China).

### 4.9. In Vivo Tests in Mice

#### 4.9.1. Animal Experimentation Design

All animal experimental procedures were approved by the Experimental Animal Ethics Committee of Changchun University of Chinese Medicine (Approval No.: 2024872, Approval Date: 31 May 2025) and were performed in strict accordance with the National Guidelines for Ethical Review of Laboratory Animal Welfare (China) and the institutional guidelines for the care and use of laboratory animals. Six-week-old male ICR mice were purchased from a commercial supplier and acclimated for one week prior to the experiment (temperature of 22 ± 2 °C, relative humidity of 40–60%, and a 12-h light/dark cycle). The mice were randomly allocated into five groups (*n* = 10 per group): a control group (normal saline), a CTX model group (40 mg/kg/d, intraperitoneal injection), and three yam protein treatment groups. The yam protein used was the total protein extract from *Dioscorea opposita cv. Lutiegun* (L-YP); for clarity, this material is hereafter referred to as YP. The dosage regimen for YP was selected based on the clinical dosage of yam documented in the Pharmacopoeia of the People’s Republic of China 2020 Edition, which was converted to a mouse equivalent dose using body surface area normalization. Accordingly, the treatment groups received YP at doses of 15 mg/kg/d (YP-L), 30 mg/kg/d (YP-M), and 45 mg/kg/d (YP-H) via oral gavage [16], the middle dose (30 mg/kg/d) represents the human-equivalent dose. The low dose (15 mg/kg/d) and high dose (45 mg/kg/d) correspond to 0.5× and 1.5× the human-equivalent dose, respectively, to allow evaluation of potential dose-dependent effects. Dosing Regimen: Except for the control group, all mice received intraperitoneal injections of CTX (40 mg/kg/d) on days 1–7. From day 8 onward, the YP-treated groups were administered the corresponding doses of YP by oral gavage for 21 days (days 8–28); the CTX model group received an equal volume of normal saline by oral gavage during the same period. The control group received normal saline by oral gavage for 28 consecutive days. Throughout the study, every effort was made to minimize animal suffering, including post-procedure monitoring and the establishment of humane endpoints (e.g., >20% weight loss); however, no animal reached these endpoints.

#### 4.9.2. Testicular and Kidney Indices

We measured and recorded the weights of both the testis and kidney. To calculate the indices for these organs, we used the following formula:

Indices (%) = (Weight of testicular or kidney)/(Body weight) × 100%

#### 4.9.3. Sperm Morphology

Sperm samples were collected from the epididymis, mechanically dispersed in saline, and filtered. Sperm smears were stained with 0.5% eosin (Shanghai Yuanye Biotechnology Co., Ltd., Shanghai, China) and observed under a light microscope (Nikon, Tokyo, Japan).

#### 4.9.4. Measurement of Serum Levels of Sex Hormones

After 28 days of treatment, blood was collected from the ocular vein and centrifuged to obtain serum, which was stored at −80 °C. Serum sex hormone levels were measured using commercial ELISA kits (Shanghai Enzyme-linked Biotechnology Co., Ltd., Shanghai, China).

#### 4.9.5. Measurement of Cytokines and Immunoglobulin in Testicular Tissue

Testes were collected to evaluate the levels of inflammatory factors in the testicular tissue. The tissues were measured, homogenized, and exposed to three freeze–thaw cycles before undergoing centrifugation. Supernatants were gathered to measure the levels of TNF-α, IL-6, TGF-β, IL-17, IgA, IgG, and IgM (Shanghai Enzyme Link Biotechnology Co., Ltd., Shanghai, China), as per the guidelines provided by the manufacturer.

#### 4.9.6. Histological Analysis

Testicular tissues were preserved using 4% paraformaldehyde. Following this, the samples underwent dehydration, were embedded in paraffin, and serial sections were prepared for hematoxylin and eosin staining. Finally, the slides were scanned with a digital microscope.

#### 4.9.7. Immunofluorescence (IF)

Testicular tissues underwent fixation, permeabilization, and blocking before being incubated overnight at 4 °C with primary antibodies, specifically anti-ZO-1 and anti-occludin. Following this, the tissues were treated with goat anti-rabbit IgG (Proteintech, Wuhan, China) conjugated to CoraLite488 for 1 h. DAPI was employed to restain the cell nuclei. Ultimately, images were obtained using an inverted fluorescence microscope (Ti2-U, Nikon, Japan), and the fluorescence intensity was assessed using ImageJ 1.8.0 software.

### 4.10. Molecular Weight Determination of Yam Protein

The molecular weights of yam proteins were analyzed by SDS-PAGE according to an optimized method based on Yang et al. [37]. Protein samples were denatured, separated on 12% gels, and stained with Coomassie Brilliant Blue G250 (Bio-Rad Laboratories, Inc., Hercules, CA, USA). Gel images were captured using an iBright CL1000 imaging system (Thermo Fisher Scientific, Waltham, MA, USA).

### 4.11. Molecular Docking and Molecular Dynamics

#### 4.11.1. Molecular Docking

The crystal structures of NF-κB p65 (PDB ID: 1VKX, resolution 2.0 Å), TLR4 (PDB ID: 2Z63), MyD88 (PDB ID: 4DOM), and NLRP3 (PDB ID: 7PZC) were retrieved from the Protein Data Bank. For p65, the subunit was extracted from the 1VKX heterodimer, and all protein structures were prepared by removing water molecules, adding polar hydrogen atoms, and assigning Gasteiger charges. Molecular docking was performed using AutoDock Vina 1.1.2. The docking grid for p65 was centered on its DNA-binding domain (center_x = 15.2, center_y = 8.7, center_z = 22.4) with dimensions of 40 × 40 × 40 Å^3^, and the exhaustiveness parameter was set to 32.

#### 4.11.2. Molecular Dynamics Simulations

Molecular dynamics simulations were carried out using GROMACS 2022.3 [38] with the Amber99sb-ildn force field. The YP2-p65 complex obtained from docking was solvated in a TIP3P water box with a 10 Å padding and neutralized by adding Na^+^ ions. The system was energy-minimized using the steepest descent algorithm (50,000 steps), followed by 100 ps of NVT equilibration and 100 ps of NPT equilibration. A 100 ns production simulation was then performed at 300 K and 1 bar with a 2 fs timestep. Trajectory frames were saved every 10 ps for analysis. Binding free energy was calculated using the gmx_MMPBSA tool with the MM-GBSA method. The generalized Born model igb = 2 was employed, and the salt concentration was set to 0.15 M. A total of 500 equally spaced frames were extracted from the last 50 ns of the 100 ns production trajectory for the calculation. The surface tension constant (γ) was set to 0.0072 kcal/(mol·Å^2^) and the non-polar solvation correction (β) to 0 kcal/mol.

### 4.12. Molecular-Level Validation by RT-qPCR

Total RNA was extracted using Trizol reagent (Takara, Frankfurt am Main, Germany), followed by reverse transcription into cDNA with a commercial kit (Takara) [16]. The target transcripts were amplified using gene-specific primers and SYBR Green fluorescence on a quantitative PCR system (Bio-Rad, Hercules, CA, USA), and their relative expression levels were subsequently calculated. The primer sequences, including those for TLR4 [39], p65 [40], MyD88 [41], NLRP3 [42], and the reference gene GAPDH, are listed in Table S2. The relative mRNA expression levels of the target genes were calculated using the 2^−ΔΔCt^ method and are presented normalized to GAPDH.

### 4.13. Statistical Analysis

For in vitro experiments, all assays were performed in three independent replicates. For in vivo experiments, each group consisted of 10 mice, and all endpoint measurements (e.g., histology, PCR, cytokine assays) were conducted on the same batch of animals. Data are expressed as mean ± standard deviation (SD) and analyzed using GraphPad Prism 10 (GraphPad Software, Boston, MA, USA) and Origin 2024 (OriginLab, Northampton, MA, USA). Multiple group comparisons were performed using one-way ANOVA followed by Tukey’s HSD test. A *p*-value of <0.05 was considered statistically significant. For histological evaluation, RT-qPCR, and cytokine assays, three mice per group were randomly selected for analysis.

## 5. Conclusions

This study is based on the traditional efficacy of yam in “tonifying the kidneys and replenishing essence” to identify its active proteins, using cyclophosphamide-induced immune testicular injury as the research subject, and explores the mechanism through which L-YP alleviates orchitis. Experiments demonstrate that L-YP possesses unique fluorescent properties and significant antioxidant activity. It effectively ameliorates testicular immune microenvironment disorders by repairing the blood–testis barrier (Occludin/ZO-1), and inhibiting the NLRP3 and TLR4/MyD88/NF-κB inflammatory pathways. By combining network pharmacology and molecular docking techniques, this study provides computational evidence that key components such as YP2 may contribute to immune regulation through predicted binding interactions (e.g., YP2-TLR4: −26.1 kcal/mol). In this study, molecular docking technology was innovatively combined with systematic experimental analysis to elucidate the molecular mechanism through which yam protein ameliorates testicular injury through the synergistic effect of the “structure–function-target”, providing a novel dietary intervention strategy for the prevention of chemotherapy-induced reproductive toxicity and highlighting the application potential of the homology of medicine and food components in the field of male reproductive health.

## Figures and Tables

**Table 1:** Analysis of Amino Acid Composition of Two Yam.

Amino Acid Type	Unit	L-YP	S-YP
Aspartic acid	mg/g	143.2370 ± 0.4248	132.3701 ± 0.1931
Glutamic acid	mg/g	164.3396 ± 0.1351	154.8903 ± 0.3747
Serine	mg/g	52.7131 ± 0.1157	56.6489 ± 0.0011
Glycine	mg/g	24.9816 ± 0.1243	24.4977 ± 0.0278
Histidine	mg/g	24.4206 ± 0.1457	10.7628 ± 0.2516
Arginine	mg/g	101.0528 ± 0.0001	97.4461 ± 0.0754
Threonine	mg/g	21.3666 ± 0.0193	20.9209 ± 0.0498
Alanine	mg/g	33.5069 ± 0.0944	32.9442 ± 0.1397
Proline	mg/g	21.5794 ± 2.9552	23.0691 ± 0.4494
Tyrosine	mg/g	30.0709 ± 0.0892	28.8158 ± 0.0374
Valine	mg/g	36.1359 ± 0.0315	35.0326 ± 0.0674
Methionine	mg/g	17.3589 ± 0.0534	14.7298 ± 0.0902
Isoleucine	mg/g	31.7786 ± 0.0148	31.0835 ± 0.0809
Leucine	mg/g	65.1879 ± 0.1809	62.5020 ± 0.0836
Phenylalanine	mg/g	45.6332 ± 0.0075	42.6772 ± 0.0782
Lysine	mg/g	66.1869 ± 0.0016	48.3122 ± 0.1569

## Data Availability

The original contributions presented in this study are included in the article/Supplementary Material. Further inquiries can be directed to the corresponding authors.

## Supplementary Materials

The following supporting information can be downloaded at: https://www.mdpi.com/article/10.3390/molecules31091387/s1. Figure S1. Extraction rate and purity of yam protein; Figure S2. Standard curve of protein molecular weight determination; Figure S3. In vitro antioxidant activity of yam protein; Figure S4. Effects of yam protein on CTX-induced testicular inflammasomes and TLR4/MyD88/NF-κB-related pathways in mice; Table S1. Secondary structure compositions of L-YP and S-YP; Table S2. Primer sequences of mRNA for RT-qPCR.; Table S3. Protein sequences and physicochemical properties; Table S4. Prediction of protein digestibility characteristics.
