# Supplementary material for: Yam-Active Protein Protects Against Cyclophosphamide-Induced Testicular Injury by Suppressing Inflammatory Responses
Source: Molecules. 2026 Apr 23;31(9):1387. doi: 10.3390/molecules31091387 (PMC13165299; doi:10.3390/molecules31091387)
Supplement: Supplementary file 1 [file molecules-31-01387-s001.zip › molecules-4233571-supplementary.pdf]

Yam-Active Protein Protects Against Cyclophosphamide-Induced Testicular Injury by Suppressing Inflammatory Responses

Jiahong Lu [1], Kaiwen Hao [1], Yuting Song [1], Jiaqi Fang [2], Boyuan Hu [1], Wei Liu [1], Ge Hui [1,*], Yunfei Xie [3,*] and Yu Zhao [1,*]

[1] Jilin Ginseng Academy, Changchun University of Chinese Medicine, Changchun 130117, China

[2] College of Food Science and Engineering, Jilin University, Changchun 130062, China

[3] School of Food Science and Technology, Jiangnan University, Wuxi 214122, China

* Correspondence: huige@ccucm.edu.cn (G.H.); xieyunfei@jiangnan.edu.cn (Y.X.); cnzhaoyu1972@126.com (Y.Z.)

1 Methods

1.1 Construction of bioactive proteins and disease targets

PharmMapper (https://www.lilab-ecust.cn/pharmmapper/) and Swiss-Target Prediction (http://swisstargetprediction.ch/) were employed to predict the target of the yam-derived protein. In the GeneCards database (https://www.genecards.org/), “Orchitis” were used as keywords for target screening. The Venn diagram established the target gene sets among the two.

1.2 Protein–protein interaction (PPI) network

Based on publicly available database resources, protein-protein interaction (PPI) network analysis was performed on the selected targets, and network visualization was achieved using Cytoscape 3.9.0 software.

1.3 GO and KEGG pathway enrichment analysis

GO biofunctional analysis and KEGG pathway enrichment analysis were performed on common gene targets using the DAVID database (https://david.ncifcrf.gov/), with the species set to “Homo sapiens”. Because human databases are comprehensively annotated and immune pathways are highly conserved, enrichment analysis was conducted using Homo sapiens.

1.4 Western Blot Analysis of Testicular Protein Expression

Mice testicular tissues were collected, frozen in liquid nitrogen, and then homogenized and ground on ice. The mixture was lysed for 30 minutes by adding an appropriate proportion of pre-cooled lysis buffer, followed by centrifugation for 10 minutes at 4 °C in a microcentrifuge. The supernatant was then discarded. Proteins were transferred to the PVDF membrane by performing electrophoresis on an SDS-PAGE gel with a gradient ranging from 8% to 12%. After this, the PVDF membrane underwent incubation at room temperature for one hour with a 5% BSA solution. Following this step, primary and secondary antibodies were introduced to the membrane, which was then subjected to multiple washing steps. Target proteins were then detected using an enhanced chemiluminescence (ECL) kit, with actin serving as an internal reference. Quantitative analysis was conducted using the AIWBwellTM analysis software.

2 Results

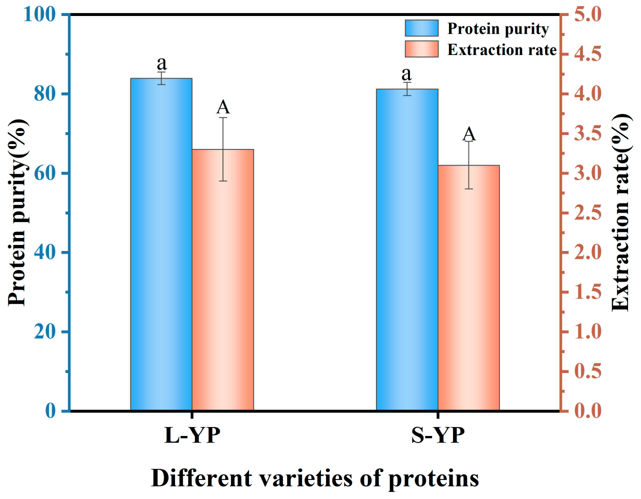

Figure.S1 Extraction rate and purity of yam protein

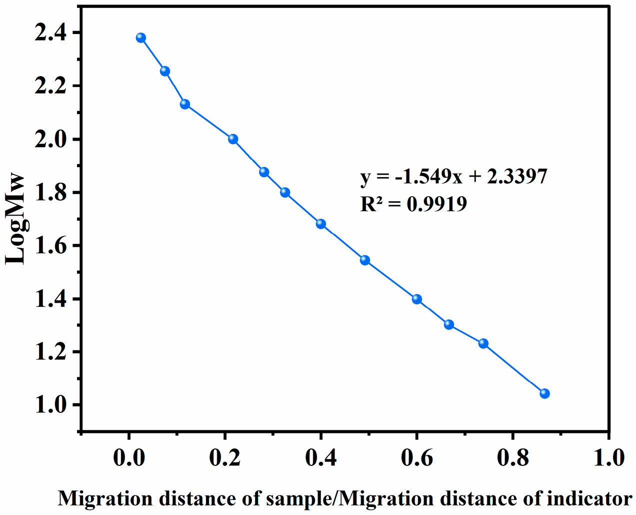

Figure.S2 Standard curve of protein molecular weight determination

Table S1 Secondary structure compositions of L-YP and S-YP

Sample	α-Helix (%)	β-Sheet (%)	β-Turn (%)	Random coil (%)
L-YP	21.27906641	26.04487077	30.09355289	22.58250993
S-YP	21.28719349	26.08541691	30.30923412	22.31815548

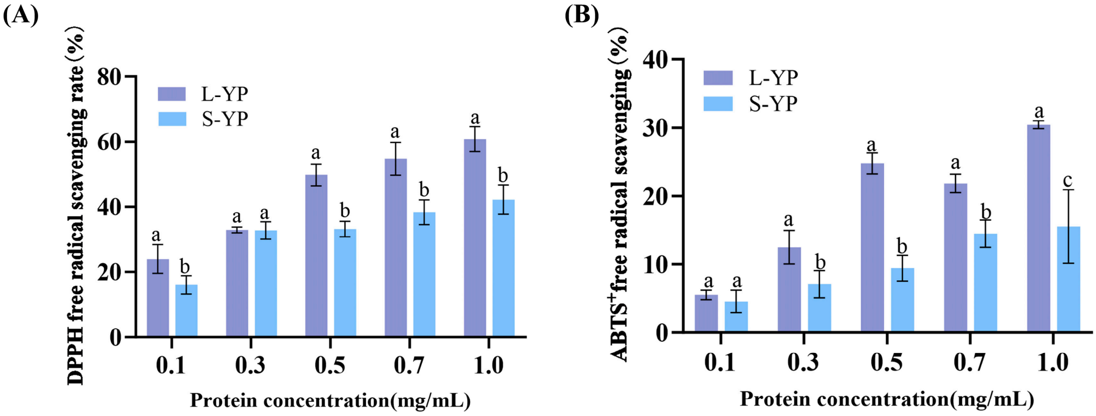

Figure.S3 *In vitro* antioxidant activity of yam protein. (A) DPPH radical scavenging activity; (B) ABTS radical scavenging activity.

Table S2. Primer sequences of mRNA for RT-qPCR.

Genes	Primer sequence (5’-3’)
TLR4	F: CAGCAAAGTCCCTGATGACA
	R: CCTGGGGAAAAACTCTGGAT
P65	F: ACACCTTCCCAGCATCCCTCAG
	R: CTTCCGACAGCGTGCCTTCC
MyD88	F: TGGCATGCCTCCATCATAGTTAACC
	R: GTCAGAAACACACACCATGC
NLRP3	F: CCCTTGGAGACACAGGACTC
	R: GAGGCTGCAGTTGTCTAATTCC
GAPDH	F: AGGTCGGTGTGAACGGATTTG
	R: GGGGTCGTTGATGGCAACA

Table S3 Protein sequences and physicochemical properties

Number	Name	Amino acid sequence	Number of amino acids	Molecular weight	Hydrophobicity	Instability factor
YP1 (SMT1-1)	Cycloartenol 24-C-methyltransferase	MIAISIGIKPCGWFGEHLRLCQGTIAIWRSVIL<br>RDGQTNFTILVLMLLFSIMQGDFMKMSFPDN<br>TYDAVYAIEATCHAPDALGCYKEIYRVLKP<br>GQCFAAYEWCMTDHYDPNNETHKKIKAEIE<br>LGNGLPDVRSTAQCLEALKQAGFEVIWEKD<br>LAADSPVTWYLPLDTSRFSITSFRLTAFGRLI<br>TRTMVKTLEFVGLAPAGSERVSSFLEKAAEG<br>LVEGGRKEIFTPMYFFLVRKPLSDS*	242	27.23	0.054	35.35
YP2 (OAT)	Mitochondrial Ornithine Aminotransferase	MASSKRPLQCLLKRALGTRSFCALPKKSSAS<br>PSSSSEELMRMEHEYSAHNYHPIPMVFSQAK<br>GTCIWDPEGHKYIDFLSAYSAVNQGHCHPK<br>VMKALVEQAERLTLSSRAFYNDKFPVLAEY<br>LTHLFGYDMVLPMNTGAEGVETALKLARK<br>WGYEKKKIPKNEAIIVSCCGCFHGRTLGAIS<br>MSCDNDATRGFGPLVPGHLKVDFGDVDALE<br>KIFKDHGDQICGFLFEPIQGEAGVCILINFVLD<br>AY*	238	27.37	-0.142	32.38
YP3 (DIOA3)	dioscorin precursor	MSSSILLHLLLLSSLLFSCLANVEDEFSYIEGN<br>PNGPENWGNLKPEWETCGKGMEQSPIQLRD<br>NRVIFDQTLGRLRRNYRAVDARLRNSGHDV<br>LVEFKGNAGSLSINRVEYQLKRIHFHSPSEHA<br>LNGERYDLEVQMVHESQDQRRAVTAILFRF<br>GRSDPFLSDLEDFISQISNSEKNEVDAGVVDP<br>RQLLQFDDPAYYRYMGSFTAPPCTEDITWT<br>VIKKLGTVSPKQVLMLKQAVNENAINNARP<br>LQPQKFRTVFFYPRQKSNHGAI*	269	30.79	-0.498	47.75
YP4 (DB1)	Tuber Agglutinin 1	MAIPGAFVSLSLALLLVASPLCCMADFILYS<br>GDSLRSGQSLTYSSYTYIMQDDCNLVLYDN<br>GKAIWASGTNGRGTNCRVTMQSDGNLVVY<br>TSNNNAVWASNTNVGNGHYVCILQRDRNV<br>VIYGGARWATNTNTVGVSGGMFIESKATIFG<br>SLPANETTAEAKAARISMVVNK*	160	18.345	0.117	27.79
YP5 (HTR4)	Histone-like Protein	MARTKQTARKSTGGKAPRKQLATKAARKS<br>APTTGGVKKPHRYRPGTVALREIRKYQKSTE<br>LLIRKLPFQRLVREIAQDFKTDLRFQSHAVLA<br>LQEAAEAYLVGLFEDTNLCAIHAKRVTIMPK<br>DIQLARRIRGERA*	102	13.643	-0.602	38.38
YP6 (ATS3A)	Embryo-Specific Protein ATS3A	MISIAFGDAYRNEVYAPRLDDPSSGAFERCS<br>TDTFKMQGPCGYGVCYLYVRRDGWDGWTP<br>EWVQILETSYHRSVNFYYGSPIPNGVWFGFN<br>NCPRLVTRPTGGAQLVSRMRM*	100	12.81	-0.387	31.93

2.1 Prediction of protein digestibility characteristics

Bioinformatic characterization predicted YP1 and YP4 to be hydrophilic, while the others were hydrophobic. YP3 showed a predicted instability coefficient >40, suggesting structural liability. In silico gastrointestinal digestion analysis indicated YP3 had the highest number of predicted cleavage sites (156), whereas YP6 was the most stable (65 sites), yielding a digestibility order of YP3 > YP1 > YP2 > YP4 > YP5 > YP6 (Table S4). These analyses of physicochemical and digestibility properties provide insights into the potential functional stability and bioavailability of the constituent proteins.

Table S4 Prediction of protein digestibility characteristics

**Number**	**Pepsin（pH1.3）**	**Pepsin（pH>2）**	**Trypsin**	**Total digestion site**
YP1	57	76	22	155
YP2	47	61	26	134
YP3	57	70	29	156
YP4	32	50	11	93
YP5	22	24	29	75
YP6	20	35	10	65

2.2 Protein expression Analysis of the Regulatory Effect of YP on Core proteins in Testicular Inflammation

The expression of NLRP3 and proteins associated with the TLR4/MyD88/NF-κB pathway in CTX-induced mice testes was validated, with results presented in Figure S4 (A-E). Compared with the Control group, the expression of NLRP3, TLR4, MyD88, and p65 in mice testes was significantly increased following CTX induction ($P < 0.05$). After administration of YP, these increases were significantly reversed ($P < 0.05$). These findings indicated that YP ameliorated CTX-induced testicular inflammation by downregulating the elevated expression of NLRP3 and other inflammation-related proteins caused by CTX.

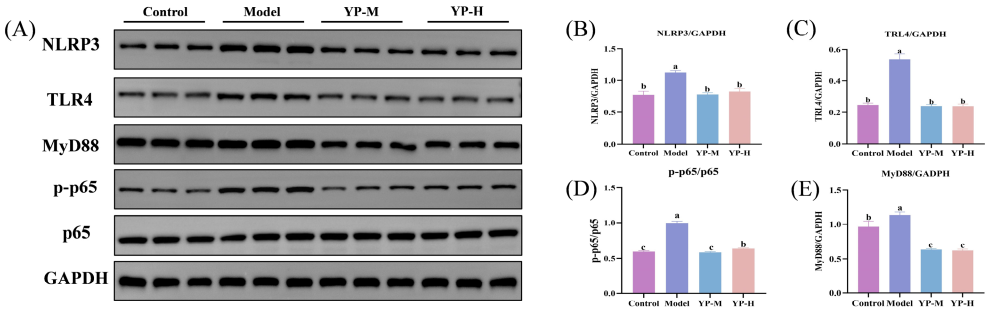

Figure S4. Effects of yam protein on CTX-induced testicular inflammasomes and TLR4/MyD88/NF-κB-related pathways in mice. (A-E) Inflammasome and TLR4/MyD88/NF-κB-related protein expression. Different lowercase letters indicate significant differences between groups, *P*<0.05.
